# Supplementary material for: Prediction of Bending Properties for 3D-Printed Carbon Fibre/Epoxy Composites with Several Processing Parameters Using ANN and Statistical Methods
Source: Polymers (Basel). 2022 Sep 4;14(17):3668. doi: 10.3390/polym14173668 (PMC9459871; doi:10.3390/polym14173668)
Supplement: Supplementary file 1 [file polymers-14-03668-s001.zip › polymers-1861082-supplementary.pdf]

*Article*

# Prediction of bending properties for 3D-printed carbon fiber/epoxy composites with several processing parameters using ANN and statistical methods

**Francisco Monticeli [1,*], Roberta Neves [2], Heitor Ornaghi [3], Humberto Almeida [4,*]**

[1] Department of Aeronautical Engineering, Technological Institute of Aeronautics (ITA), São José dos Campos, Brazil
[2] PPGE3M, Federal University of Rio Grande do Sul, Porto Alegre, RS, Brazil
[3] Mantoflex Indústria de Plásticos Ltda, Caxias do Sul, Brazil
[4] Advanced Composites Research Group, School of Mechanical and Aerospace Engineering, Queen's University Belfast, Belfast, UK

* Correspondence: humberto.almeida@qub.ac.uk (HA) and fmonticeli@ita.br (FM)

1. ANN prediction procedure

Figure S1 presents the performance of ANN based on mean square errors (MSE), in which the algorithm stops training when generalization ceases to improve according to the decrease of MSE.

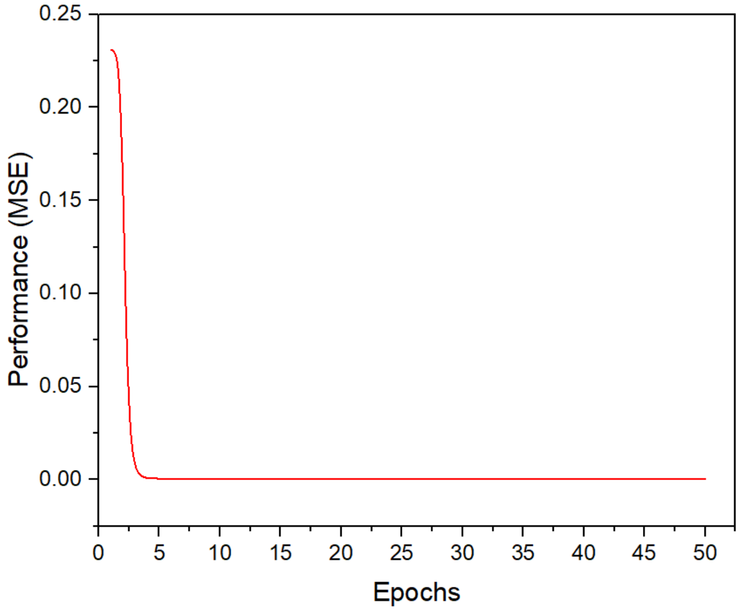


**Figure S1.** Performance of the neural network structure (based on MSE).

Figure S2 illustrate the sequence of input parameters using the OriginLab® software. Point 1 indicates the data tab; meanwhile, point 2 exhibits the experimental data of the process parameters and the chosen result (flexural strength, for instance). It is important to inform that the input data must not be modified. Point 3 indicates the chosen parameters levels in which the prediction will be generated. These parameters could be others not used experimentally.

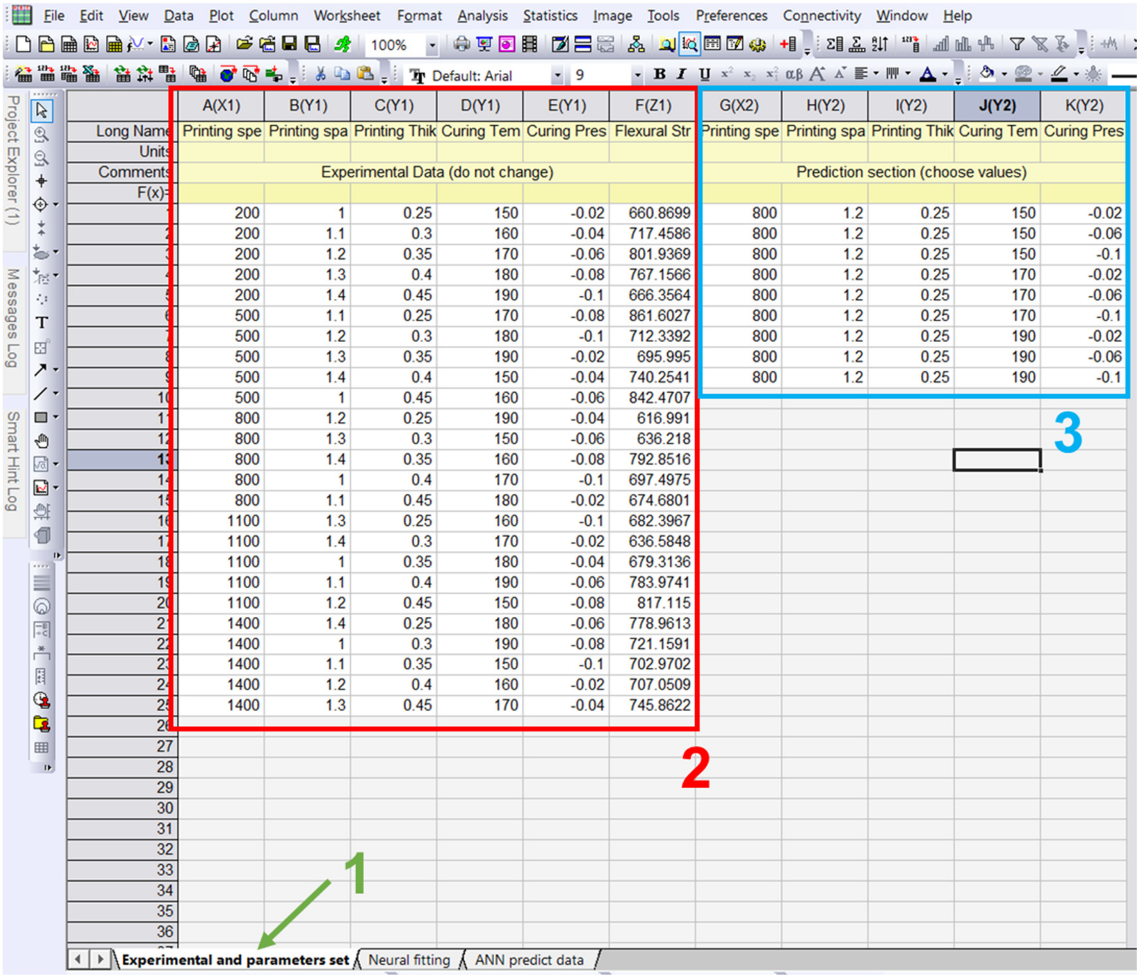

| | A(X1) | B(Y1) | C(Y1) | D(Y1) | E(Y1) | F(Z1) | G(X2) | H(Y2) | I(Y2) | J(Y2) | K(Y2) |
|---|---|---|---|---|---|---|---|---|---|---|---|
| Long Name | Printing spe | Printing spa | Printing Thik | Curing Tem | Curing Pres | Flexural Str | Printing spe | Printing spa | Printing Thik | Curing Tem | Curing Pres |
| Units | | | | | | | | | | | |
| Comments | Experimental Data (do not change) | | | | | | Prediction section (choose values) | | | | |
| F(x)= | | | | | | | | | | | |
| 1 | 200 | 1 | 0.25 | 150 | -0.02 | 660.8699 | 800 | 1.2 | 0.25 | 150 | -0.02 |
| 2 | 200 | 1.1 | 0.3 | 160 | -0.04 | 717.4586 | 800 | 1.2 | 0.25 | 150 | -0.06 |
| 3 | 200 | 1.2 | 0.35 | 170 | -0.06 | 801.9369 | 800 | 1.2 | 0.25 | 150 | -0.1 |
| 4 | 200 | 1.3 | 0.4 | 180 | -0.08 | 767.1566 | 800 | 1.2 | 0.25 | 170 | -0.02 |
| 5 | 200 | 1.4 | 0.45 | 190 | -0.1 | 666.3564 | 800 | 1.2 | 0.25 | 170 | -0.06 |
| 6 | 500 | 1.1 | 0.25 | 170 | -0.08 | 861.6027 | 800 | 1.2 | 0.25 | 170 | -0.1 |
| 7 | 500 | 1.2 | 0.3 | 180 | -0.1 | 712.3392 | 800 | 1.2 | 0.25 | 190 | -0.02 |
| 8 | 500 | 1.3 | 0.35 | 190 | -0.02 | 695.995 | 800 | 1.2 | 0.25 | 190 | -0.06 |
| 9 | 500 | 1.4 | 0.4 | 150 | -0.04 | 740.2541 | 800 | 1.2 | 0.25 | 190 | -0.1 |
| 10 | 500 | 1 | 0.45 | 160 | -0.06 | 842.4707 | | | | | |
| 11 | 800 | 1.2 | 0.25 | 190 | -0.04 | 616.991 | | | | | |
| 12 | 800 | 1.3 | 0.3 | 150 | -0.06 | 636.218 | | | | | |
| 13 | 800 | 1.4 | 0.35 | 160 | -0.08 | 792.8516 | | | | | |
| 14 | 800 | 1 | 0.4 | 170 | -0.1 | 697.4975 | | | | | |
| 15 | 800 | 1.1 | 0.45 | 180 | -0.02 | 674.6801 | | | | | |
| 16 | 1100 | 1.3 | 0.25 | 160 | -0.1 | 682.3967 | | | | | |
| 17 | 1100 | 1.4 | 0.3 | 170 | -0.02 | 636.5848 | | | | | |
| 18 | 1100 | 1 | 0.35 | 180 | -0.04 | 679.3136 | | | | | |
| 19 | 1100 | 1.1 | 0.4 | 190 | -0.06 | 783.9741 | | | | | |
| 20 | 1100 | 1.2 | 0.45 | 150 | -0.08 | 817.115 | | | | | |
| 21 | 1400 | 1.4 | 0.25 | 180 | -0.06 | 778.9613 | | | | | |
| 22 | 1400 | 1 | 0.3 | 190 | -0.08 | 721.1591 | | | | | |
| 23 | 1400 | 1.1 | 0.35 | 150 | -0.1 | 702.9702 | | | | | |
| 24 | 1400 | 1.2 | 0.4 | 160 | -0.02 | 707.0509 | | | | | |
| 25 | 1400 | 1.3 | 0.45 | 170 | -0.04 | 745.8622 | | | | | |



**Figure S2.** Experimental and prediction parameter set.

Figure S3 exhibits the data analysis of ANN application at tab in Point 4, presenting the linear regression, residual plot, error, linearity coefficient, among other parameters.

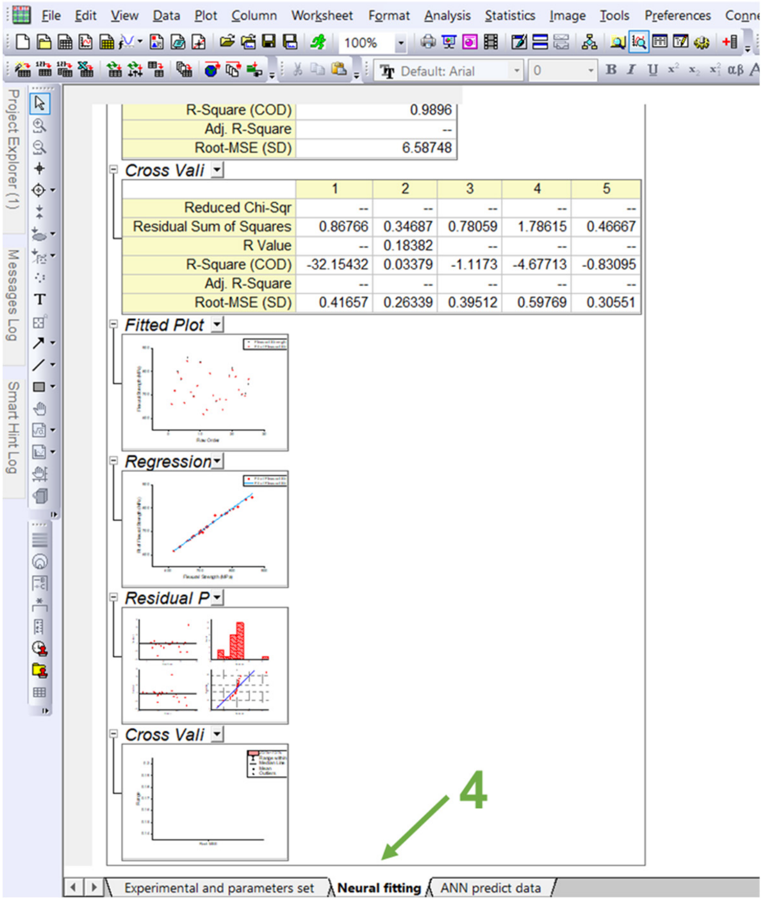


**Figure S3.** Neural network fit.

Figure S4 shows the results of ANN (tab in Point 5). Point 6 shows the experimental and prediction response. Point 7 exhibits the prediction data response using the parameter added (desired parameters according to the user) at Figure S2 Point 2, which also could be others not carried out experimentally.

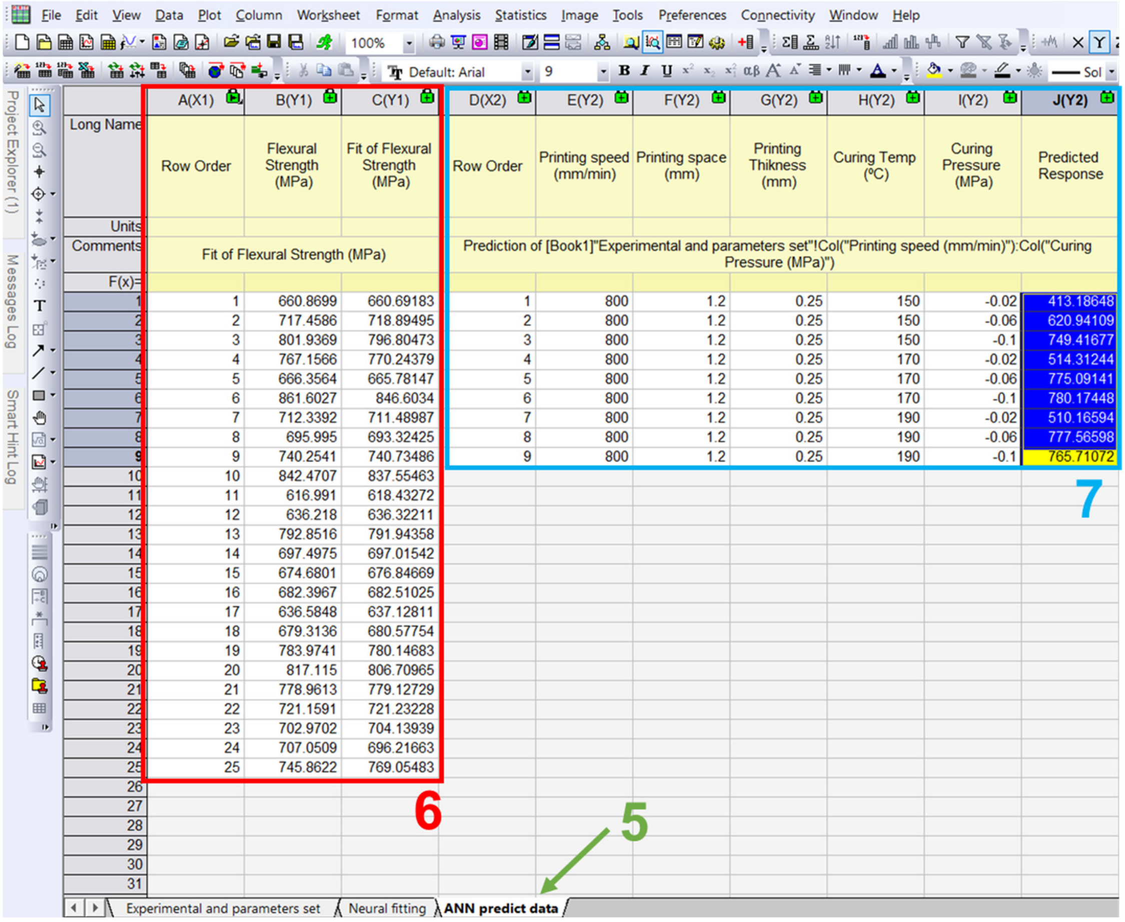


**Figure S4.** Neural network prediction data.

2. Surface response methodology

This section presents different possibilities of additive manufacturing parameters and respective trend on mechanical behavior (flexural strength – σ MPa, modulus – E GPa, and strain – s %), based on surface response methodology. Each RSM Figure is followed by the descriptive Equation (S3), in which printing speed – $P_v$ mm.min$^{-1}$, space – $P_s$ mm, thickness – $t$ mm, cure temperature – T °C, and vacuum pressure – P MPa.

*2.1. Flexural strength*

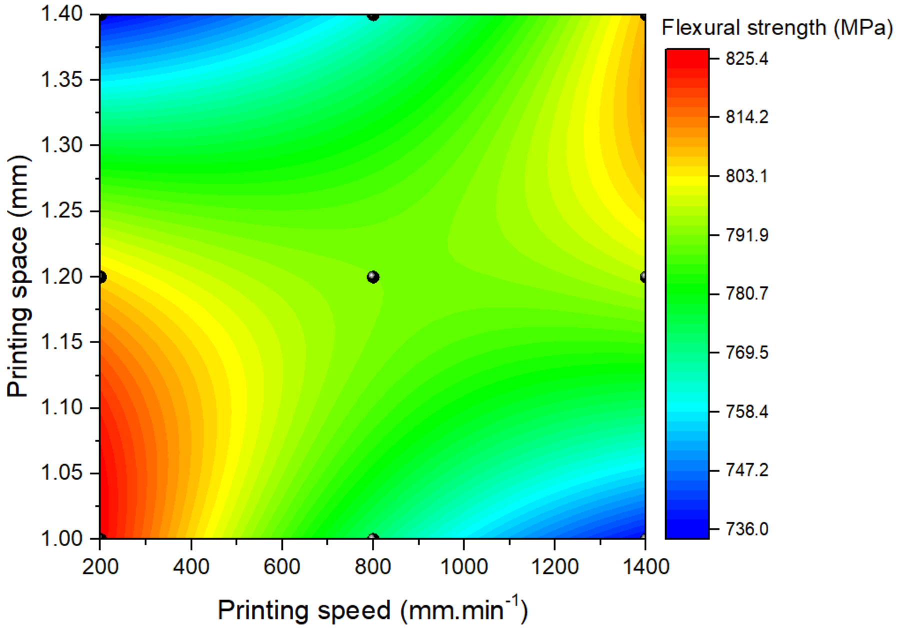


**Figure S5.** RSM of flexural strength as a function of printing space and speed.

$$\sigma = 274.9 - 0.45P_v + 1187.2P_s + 2.5 \times 10^{-5}P_v^2 - 615.2P_s^2 + 0.33P_vP_s \qquad (S1)$$

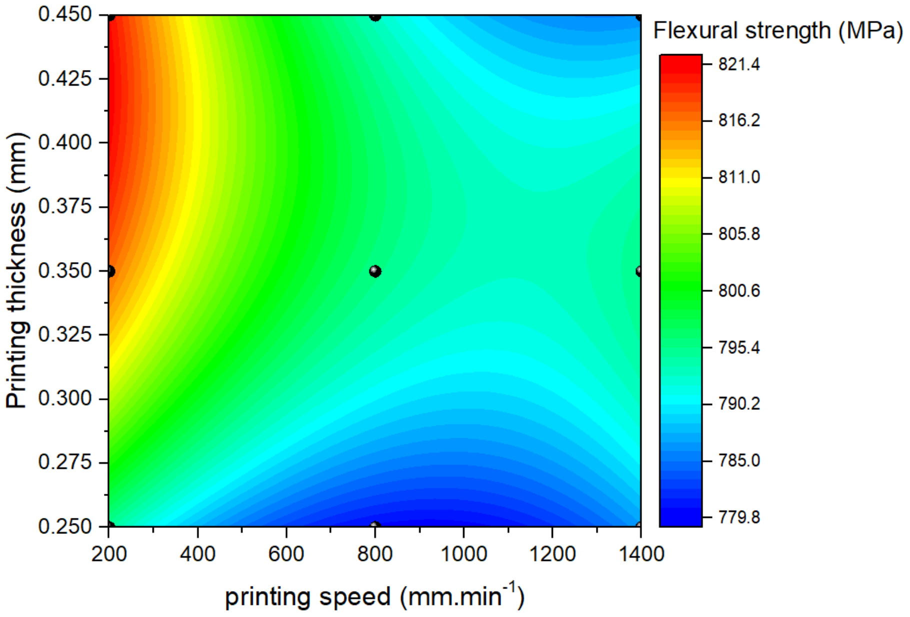


**Figure S6.** RSM of flexural strength as a function of printing thickness and speed.

$$\sigma = 665.2 - 0.025P_v + 779.7Pt + 2.8 \times 10^{-5}P_v^2 - 897.5Pt^2 - 0.11P_vPt \qquad (S2)$$

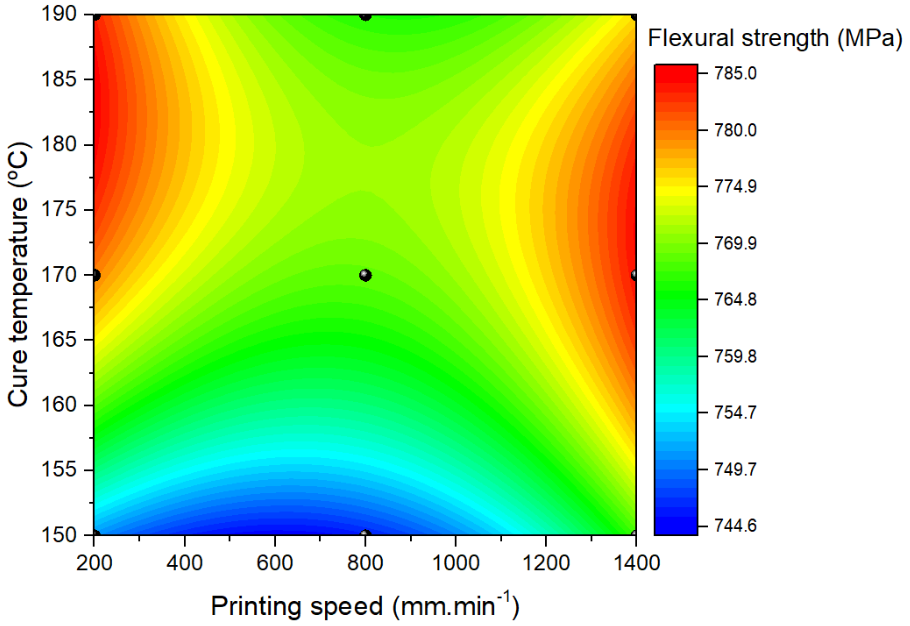


**Figure S7.** RSM of flexural strength as a function of cure temperature and printing speed.

$$\sigma = -265.4 + 0.03P_v + 11.5T + 3.6 \times 10^{-5}P_v^2 - 0.03T^2 - 5.1 \times 10^{-4}P_vT \qquad (S3)$$

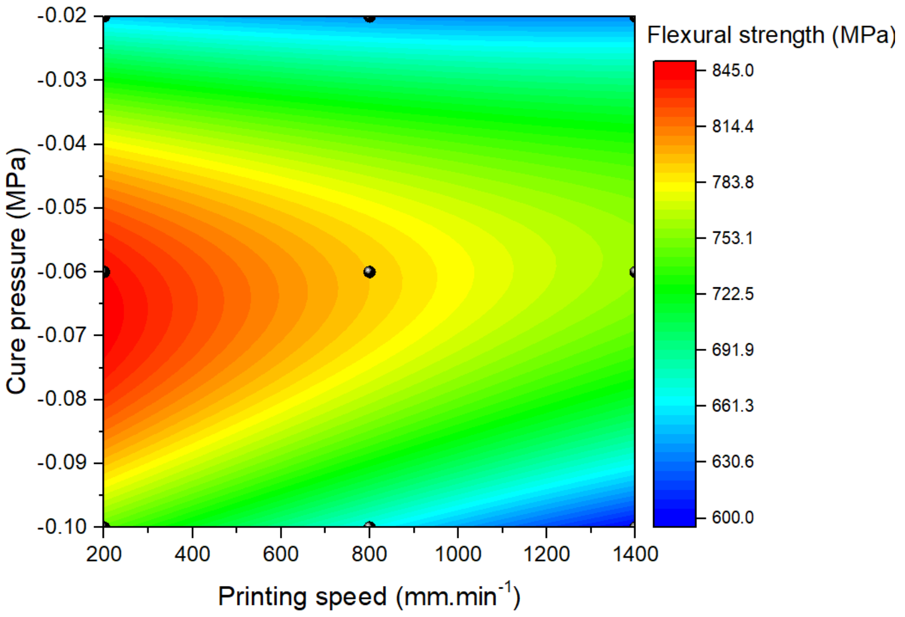


**Figure S8.** RSM of flexural strength as a function of cure pressure and printing speed.

$$\sigma = 455.4 - 0.015P_v - 12025.6P + 1.7 \times 10^{-5}P_v^2 - 88330P^2 + 1.3P_vP \qquad \text{(S4)}$$

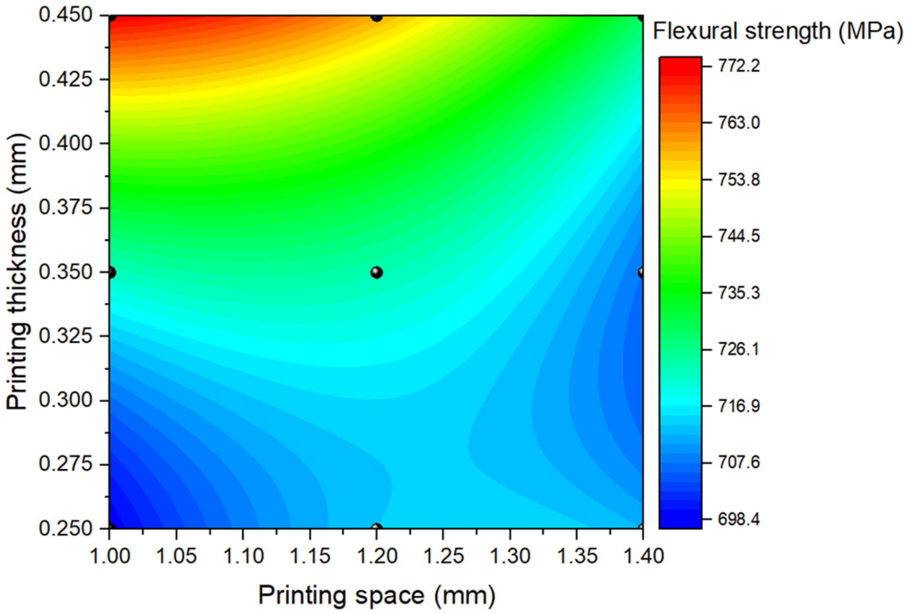


**Figure S9.** RSM of flexural strength as a function of printing thickness and space.

$$\sigma = 247 + 715P_s + 166P_t - 209P_s^2 + 1307P_t^2 - 714P_sP_t \qquad \text{(S5)}$$

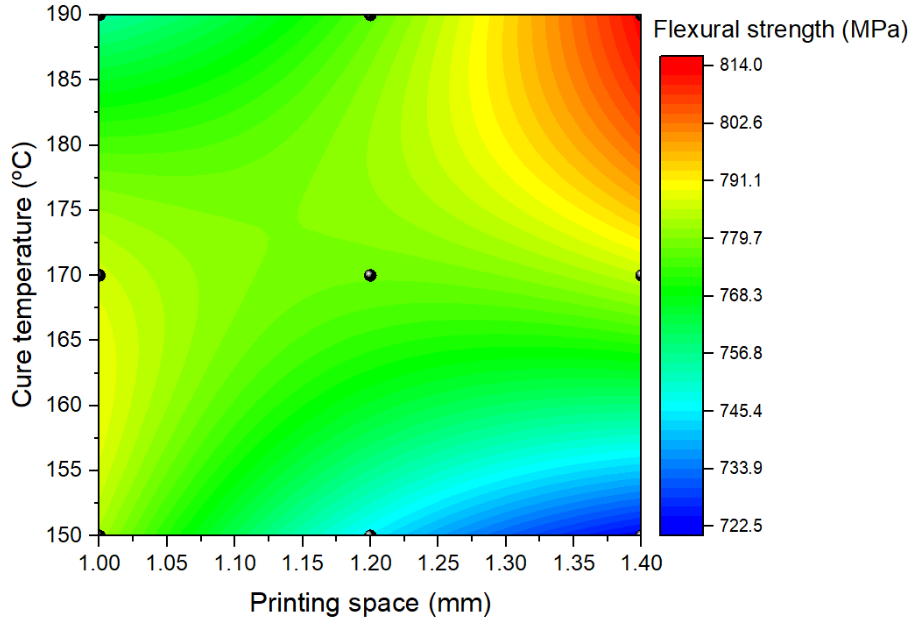


**Figure S10.** RSM of flexural strength as a function of cure temperature and printing space.

$$\sigma = 1190.8 - 1721P_s + 6.5T + 191.1P_s^2 - 0.043T^2 + 7.4P_sT \qquad \text{(S6)}$$

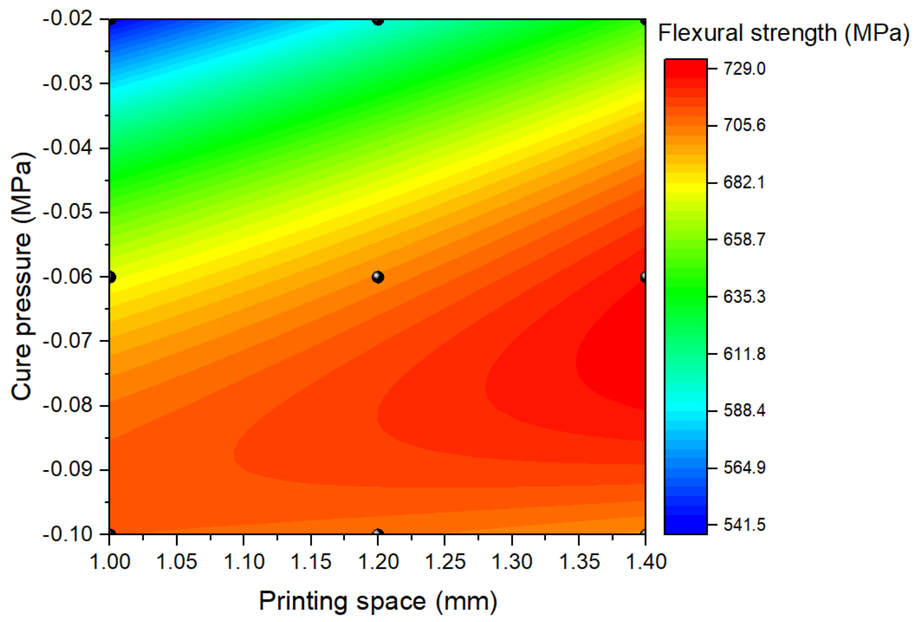


**Figure S11.** RSM of flexural strength as a function of cure pressure and printing space.

$$\sigma = 50.2 + 420.6P_s - 9538.5P - 35.5P_s^2 - 2137.7P^2 + 3582.9P_sP \quad \text{(S7)}$$

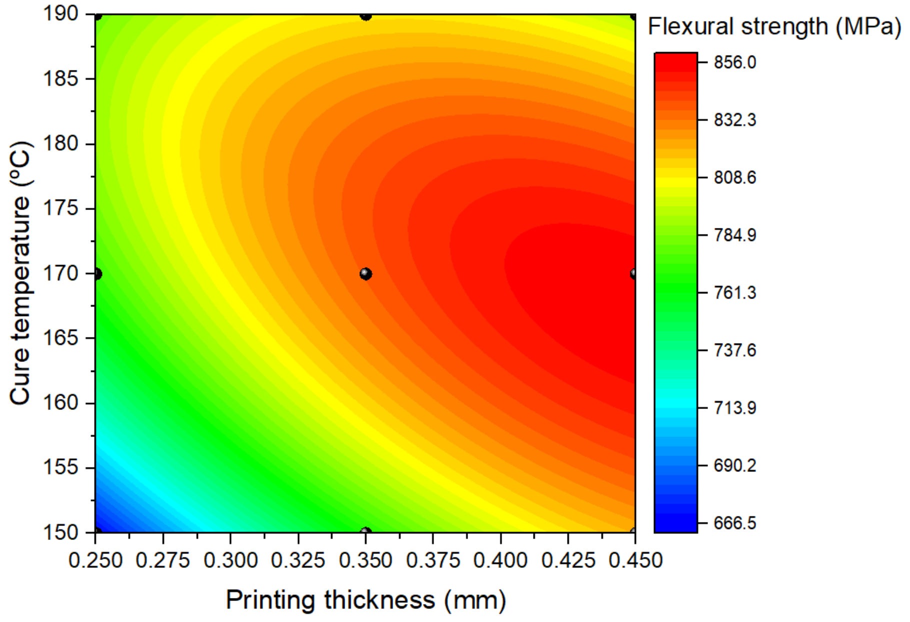


**Figure S12.** RSM of flexural strength as a function of cure temperature and printing thickness.

$$\sigma = -4299.2 + 5062.1P_t + 48.1T - 2327P_t^2 - 0.12T^2 - 17.8P_sT \quad \text{(S8)}$$

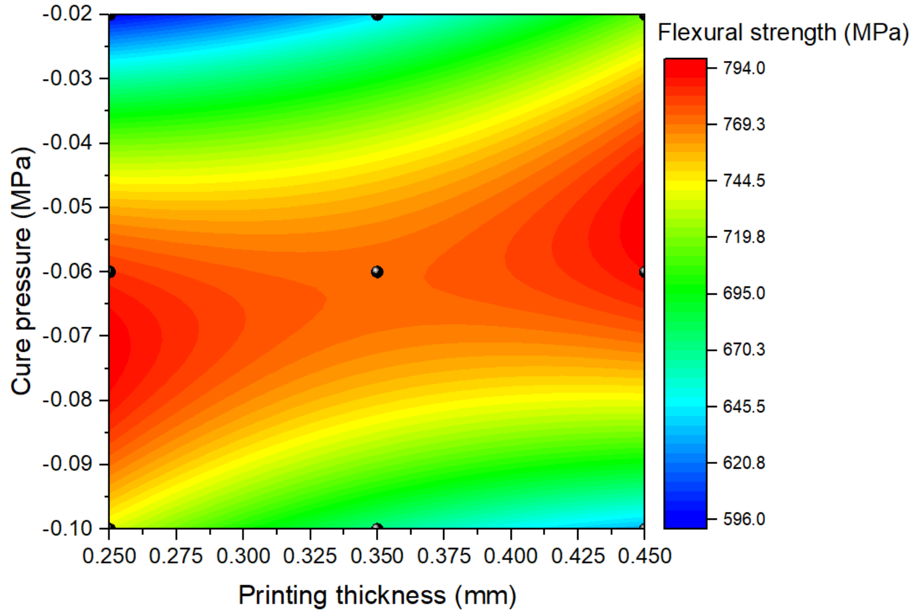


**Figure S13.** RSM of flexural strength as a function of cure pressure and printing thickness.

$$\sigma = 354 - 97.5P_t - 13933.9P + 1390.1P_t^2 - 72415.6P^2 + 13963.3P_sP \qquad (S9)$$

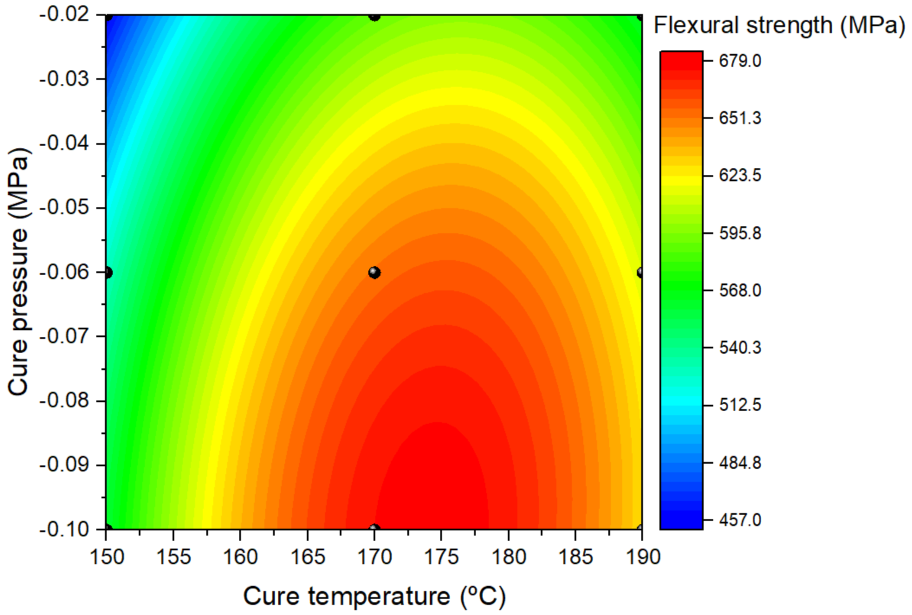


**Figure S14.** RSM of flexural strength as a function of cure pressure and temperature.

$$\sigma = -5774.6 + 71.5T - 4454.3P - 0.2T^2 - 12484.7P^2 + 11.2TP \qquad (S10)$$

*2.2. Modulus*

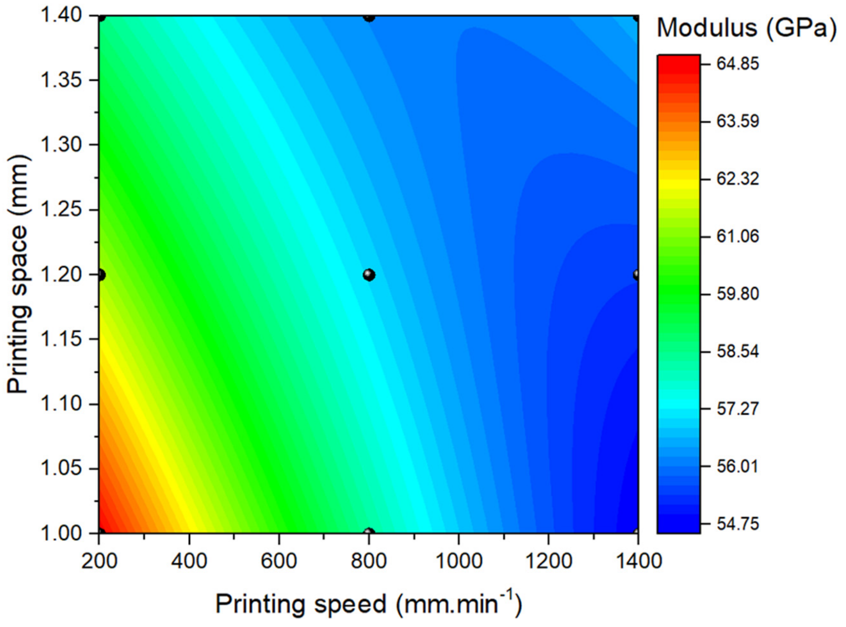


**Figure S15.** RSM of flexural modulus as a function of printing space and speed.

$$E = 96 - 0.037P_v - 35.5P_s + 3.9 \times 10^{-6}P_v^2 + 6.9P_s^2 + 0.02P_vP_s \quad \text{(S11)}$$

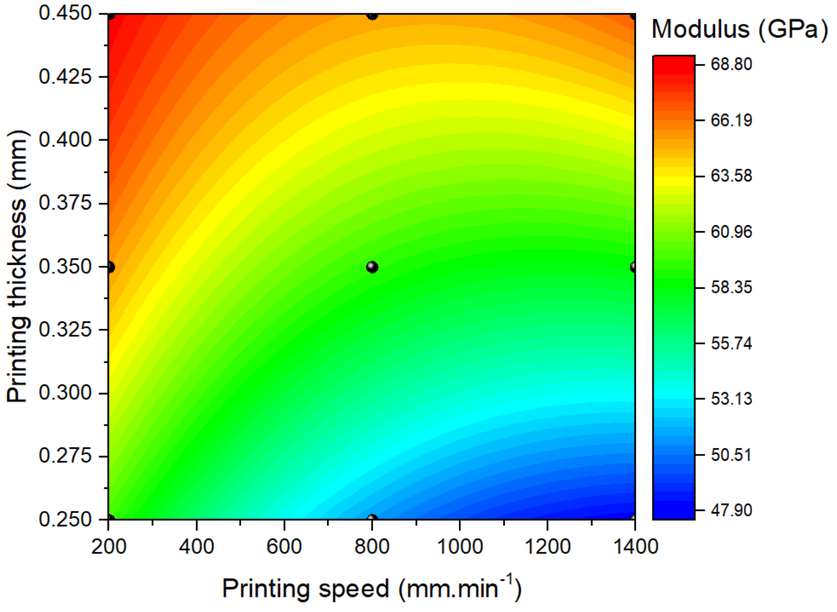


**Figure S16.** RSM of flexural modulus as a function of printing thickness and speed.

$$E = 42 - 0.03P_v + 113.9Pt + 7 \times 10^{-6}P_v^2 - 110Pt^2 + 0.04P_vPt \tag{S12}$$

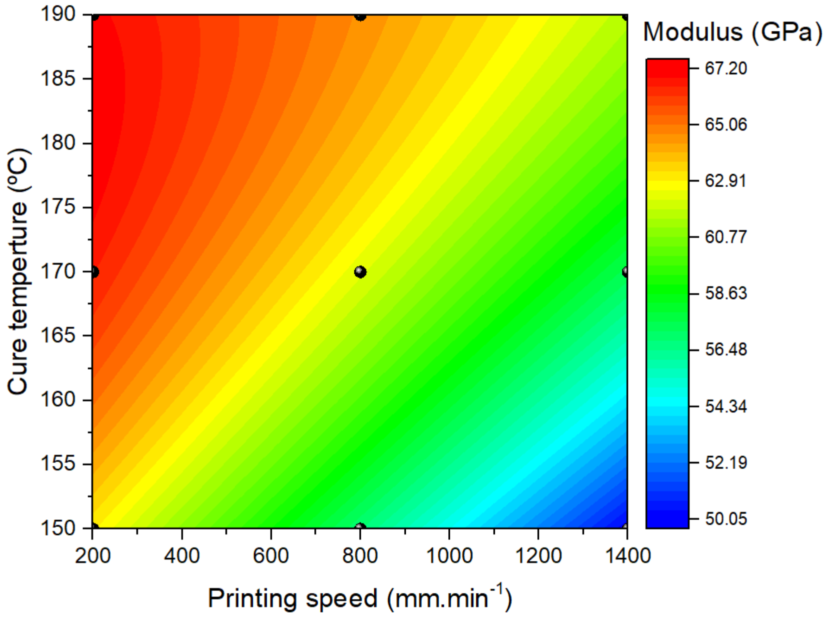


**Figure S17.** RSM of flexural modulus as a function of cure temperature and printing speed.

$$E = -55.3 - 0.03P_v + 1.4T - 9.1 \times 10^{-7}P_v^2 - 0.004T^2 + 1.6 \times 10^{-4}P_vT \tag{S13}$$

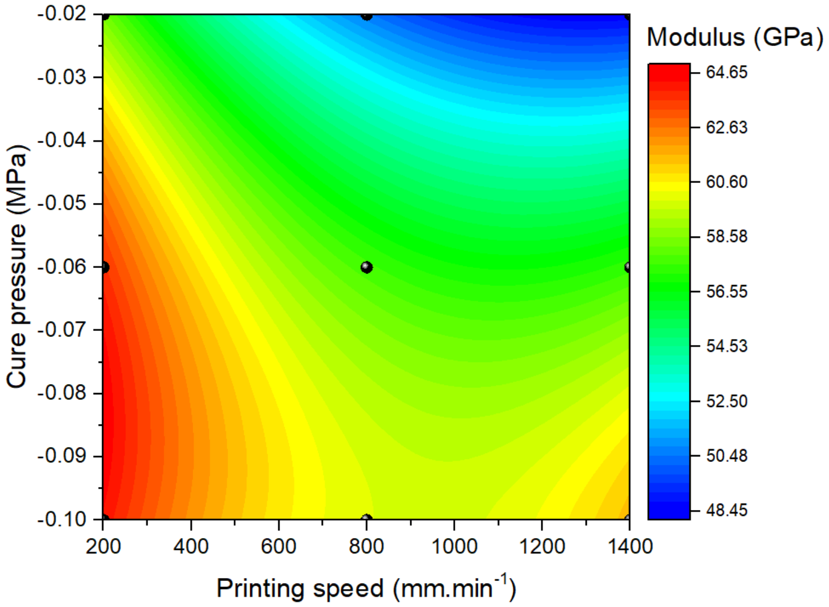


**Figure S18.** RSM of flexural modulus as a function of cure pressure and printing speed.

$$E = 58.1 - 0.023P_v - 239.3P + 8 \times 10^{-6}P_v^2 - 1511.2P^2 - 0.074P_vP \qquad (S14)$$

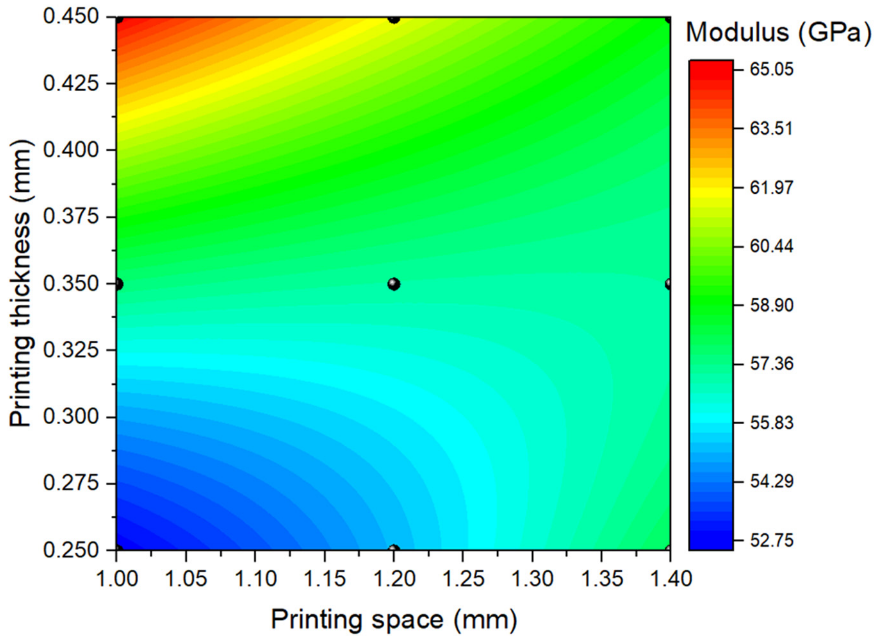


**Figure S19.** RSM of flexural modulus as a function of printing thickness and space.

$$E = 13.2 + 32.6P_s + 109P_t + 6.6P_s^2 + 133.4P_t^2 - 141.6P_sP_t \qquad (S15)$$

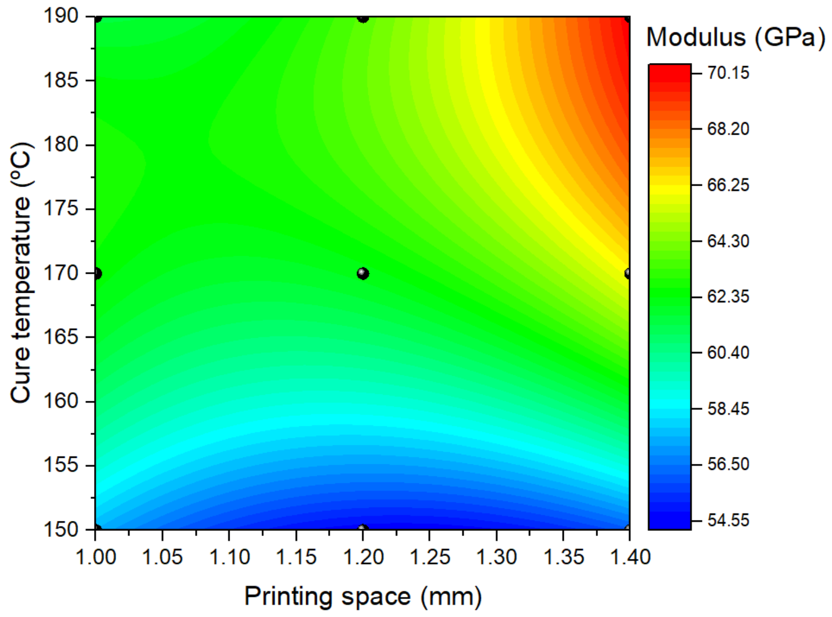


**Figure S20.** RSM of flexural modulus as a function of cure temperature and printing space.

$$E = -0.63 - 223.9P_s + 2.03T + 53.2P_s^2 - 0.007T^2 + 0.62P_sT \quad \text{(S16)}$$

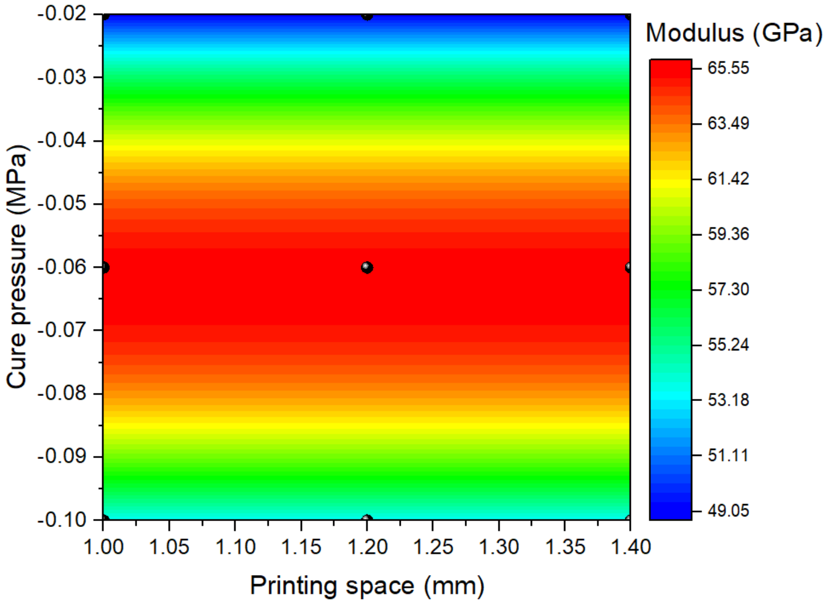


**Figure S21.** RSM of flexural modulus as a function of cure pressure and printing space.

$$E = 30 - 7 \times 10^{-13}P_s - 1118P + 3 \times 10^{-13}P_s^2 - 8864P^2 - 1.9 \times 10^{-13}P_sP \quad \text{(S17)}$$

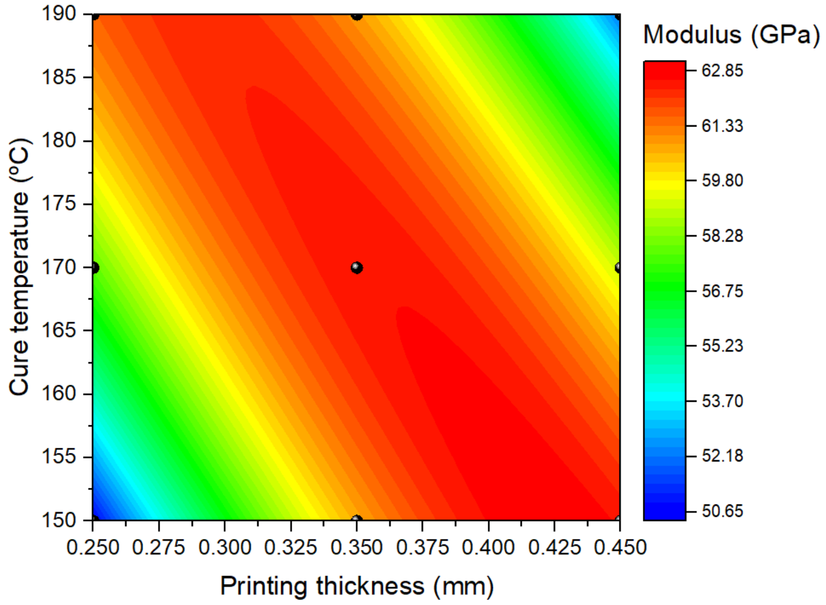


**Figure S22.** RSM of flexural modulus as a function of cure temperature and printing thickness.

$$E = -275.2 + 743.2P_t + 2.4T - 406.8P_t^2 - 0.0043T^2 - 2.66P_sT \quad \text{(S18)}$$

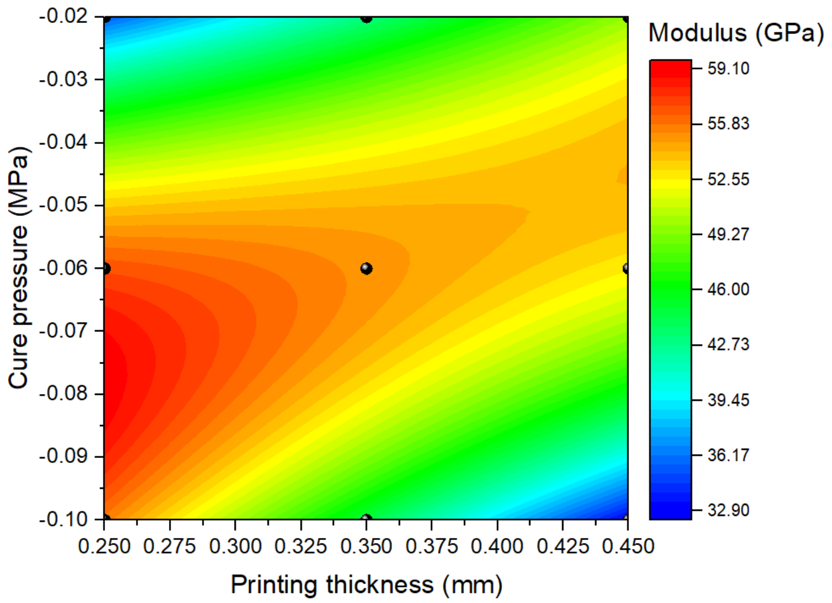


**Figure S23.** RSM of flexural modulus as a function of cure pressure and printing thickness.

$$E = -17.5 + 145.2P_t - 1673.2P - 38.5P_t^2 - 7022.9P^2 + 2316.3P_sP \quad \text{(S19)}$$

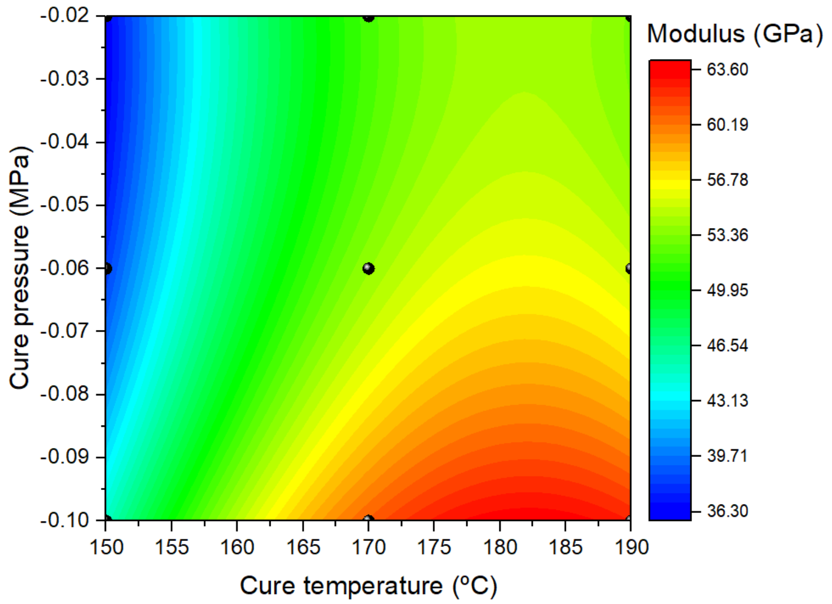


**Figure S24.** RSM of flexural modulus as a function of cure pressure and temperature.

$$E = -533.3 + 6.5T + 110.2P - 0.02T^2 + 1582.1P^2 - 0.19TP \tag{S20}$$

*2.3. Strain*

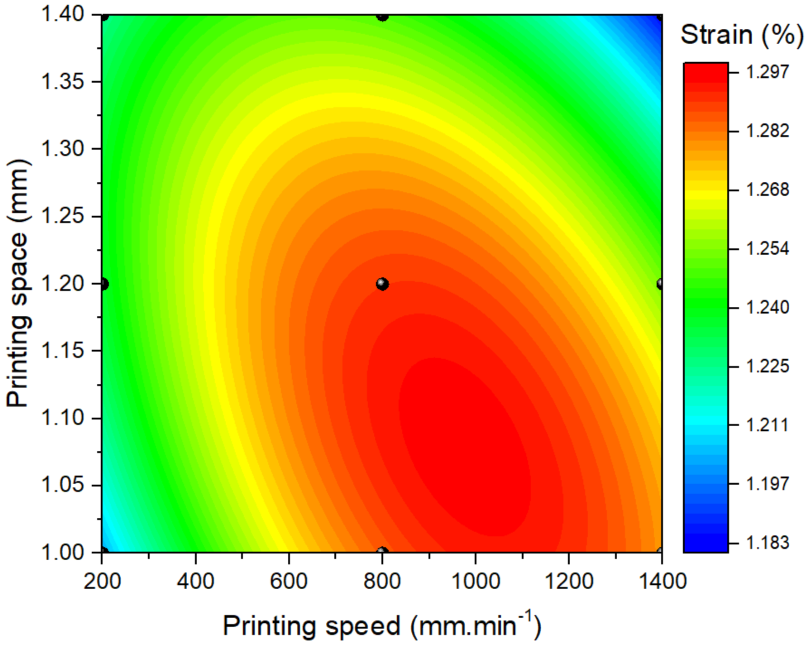


**Figure S25.** RSM of flexural strain as a function of printing space and speed.

$$s = 0.27 + 5 \times 10^{-4} P_v + 1.45 P_s - 1.2 \times 10^{-7} P_v^2 - 0.56 P_s^2 - 2.4 \times 10^{-4} P_v P_s \tag{S21}$$

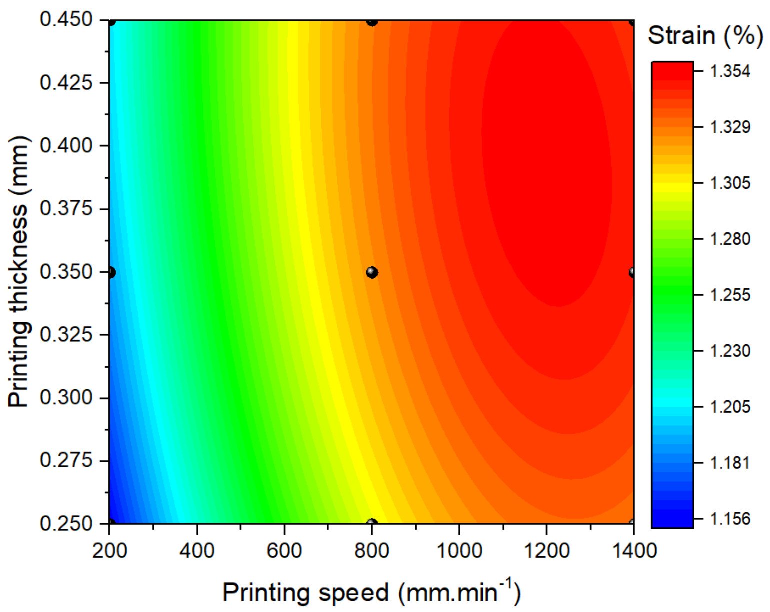


**Figure S26.** RSM of flexural strain as a function of printing thickness and speed.

$$s = 0.9 + 4 \times 10^{-4} P_v + 0.96Pt - 1.5 \times 10^{-7} P_v^2 - Pt^2 + 1.4 \times 10^{-4} P_v Pt \quad \text{(S22)}$$

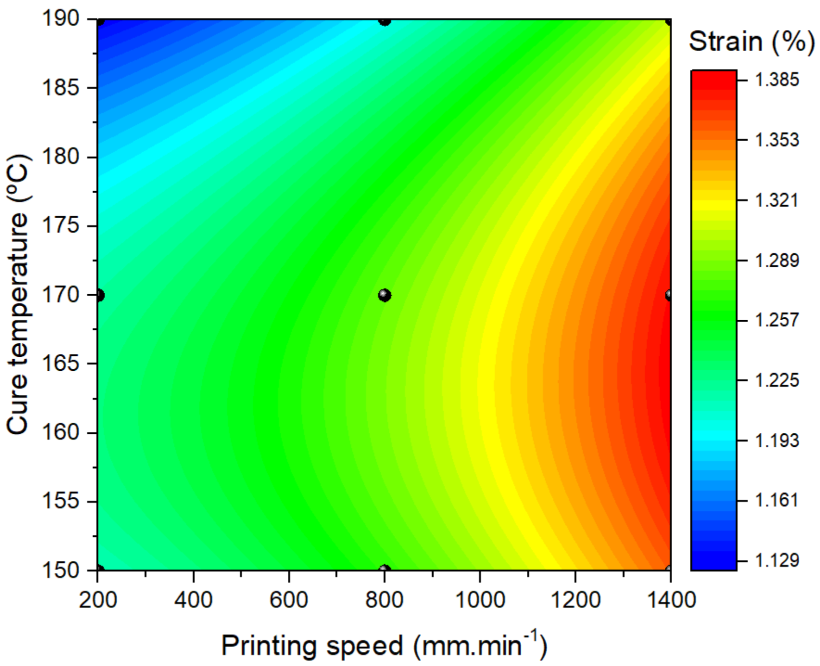


**Figure S27.** RSM of flexural strain as a function of cure temperature and printing speed.

$$s = -2 - 6 \times 10^{-5}P_v + 0.04T + 5 \times 10^{-8}P_v^2 - 10^{-4}T^2 + 6.6 \times 10^{-7}P_vT \qquad (S23)$$

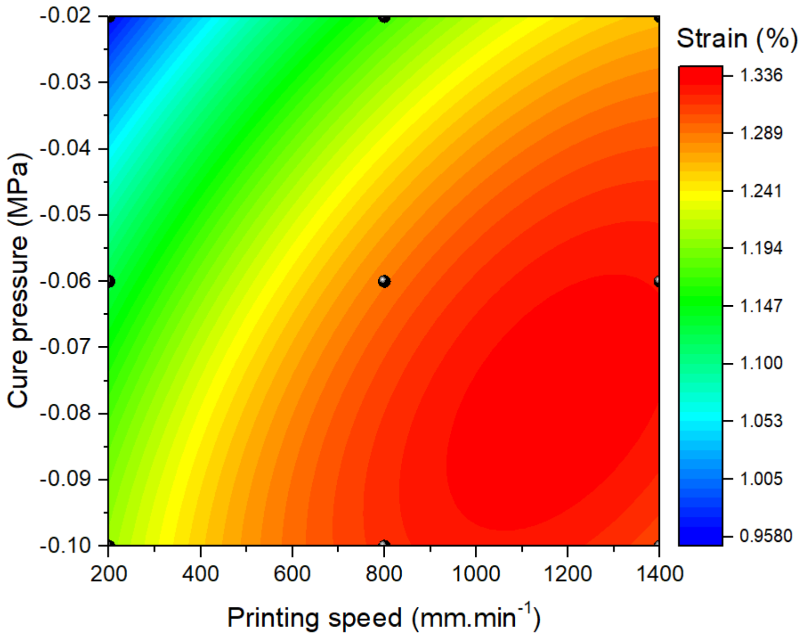


**Figure S28.** RSM of flexural strain as a function of cure pressure and printing speed.

$$s = 0.7 + 5.6 \times 10^{-4}P_v - 6.8P - 1.7 \times 10^{-7}P_v^2 - 27.5P^2 + 0.002P_vP \qquad (S24)$$

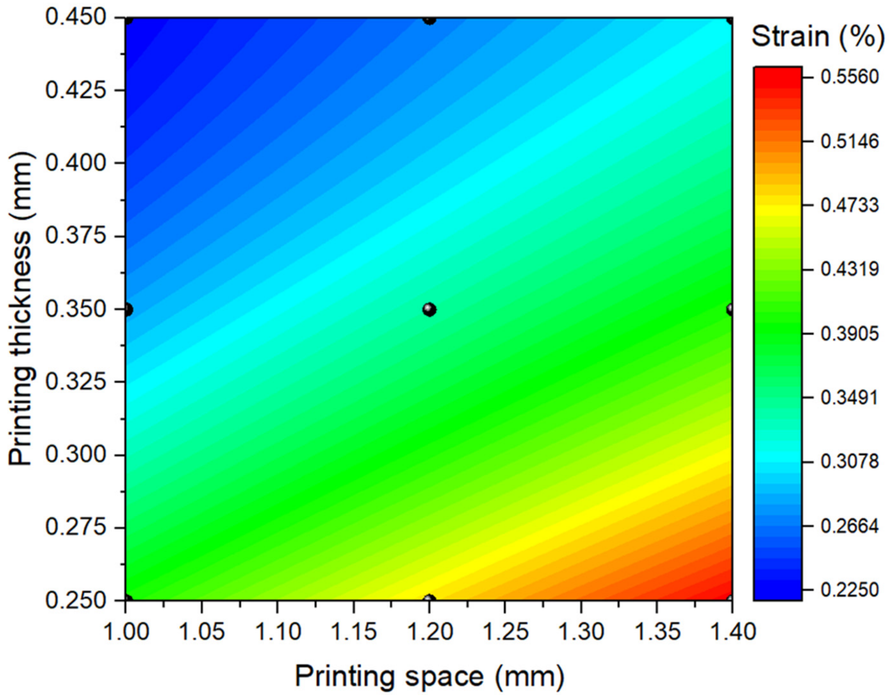

**Figure S29.** RSM of flexural strain as a function of printing thickness and speed.

$$s = 0.4 + 0.6P_s - 2.1P_t + 3.3 \times 10^{-12}P_s^2 + 3.1P_t^2 - 0.9P_sP_t \quad \text{(S25)}$$

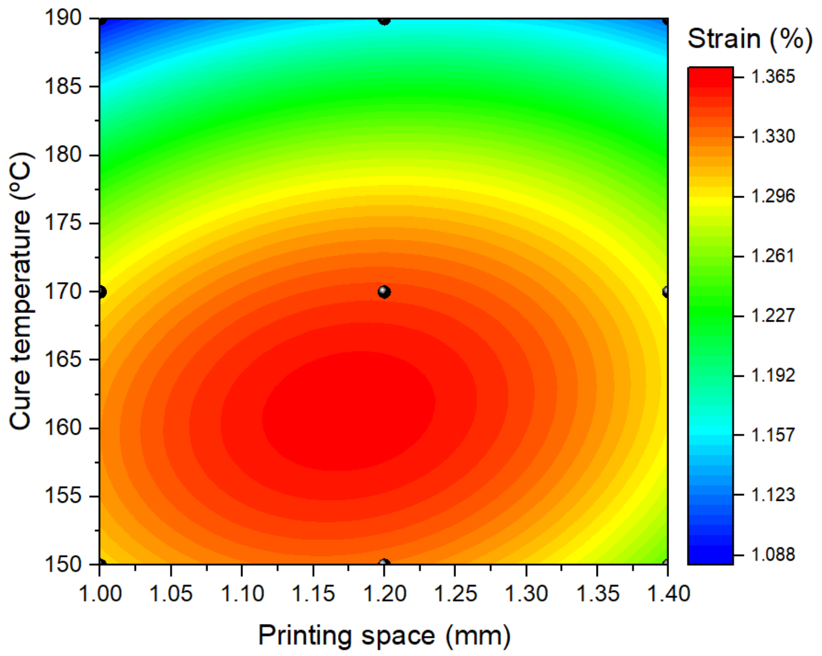


**Figure S30.** RSM of flexural strain as a function of cure temperature and printing space.

$$s = -6.1 + 2.3P_s + 0.07T - 1.3P_s^2 - 2.5 \times 10^{-4}T^2 + 0.005P_sT \quad \text{(S26)}$$

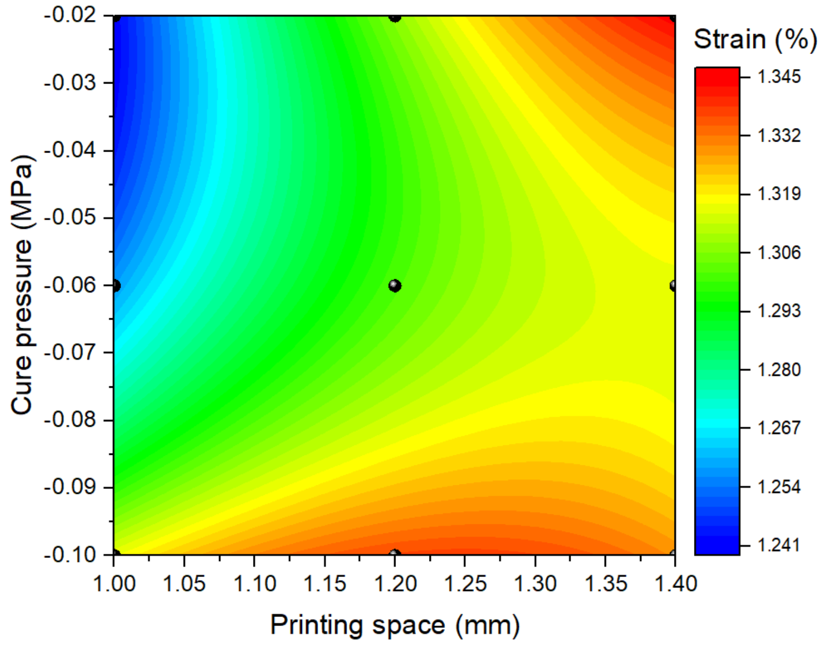


**Figure S31.** RSM of flexural strain as a function of cure pressure and printing space.

$$s = 0.45 + 1.2P_s - 2.2P - 0.4P_s^2 + 13.1P^2 + 2.83P_sP \qquad \text{(S27)}$$

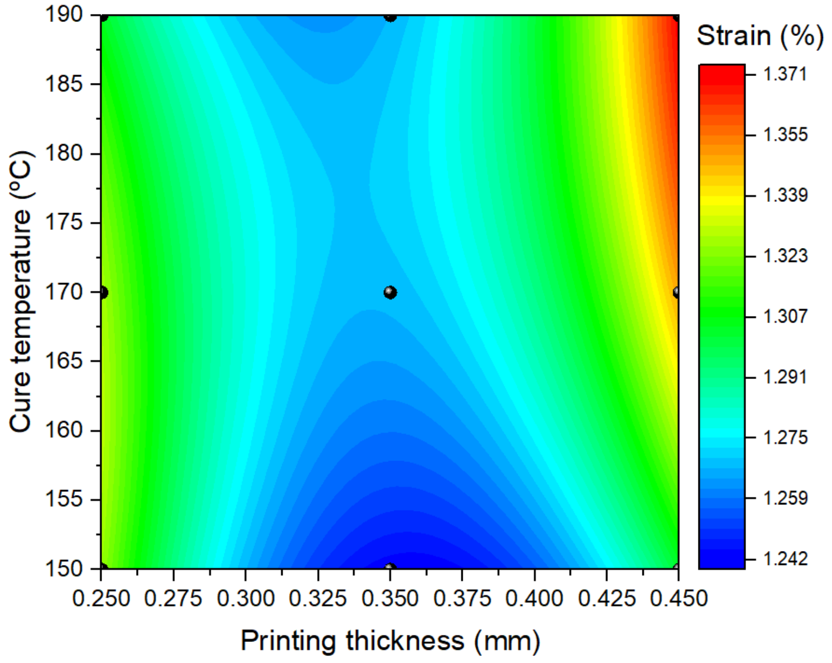


**Figure S32.** RSM of flexural strain as a function of cure temperature and printing thickness.

$$s = 1.7 - 6.7P_t + 0.007T + 7.1P_t^2 - 3.1 \times 10^{-5}T^2 + 0.012P_sT \quad (S28)$$

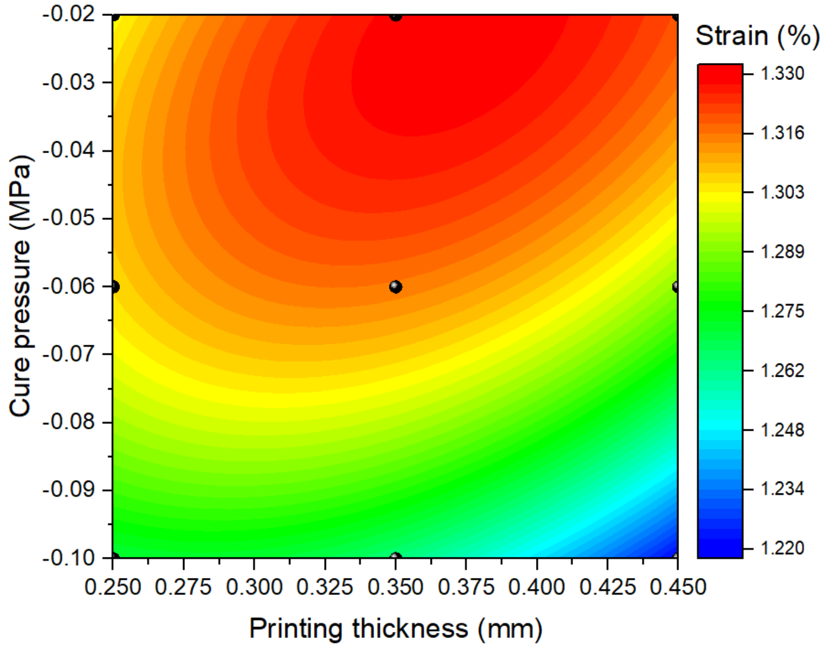


**Figure S33.** RSM of flexural strain as a function of cure pressure and printing thickness.

$$s = 1.03 + 1.4P_t - 2.2P - 1.8P_t^2 - 12.4P^2 + 4.29P_sP \quad (S29)$$

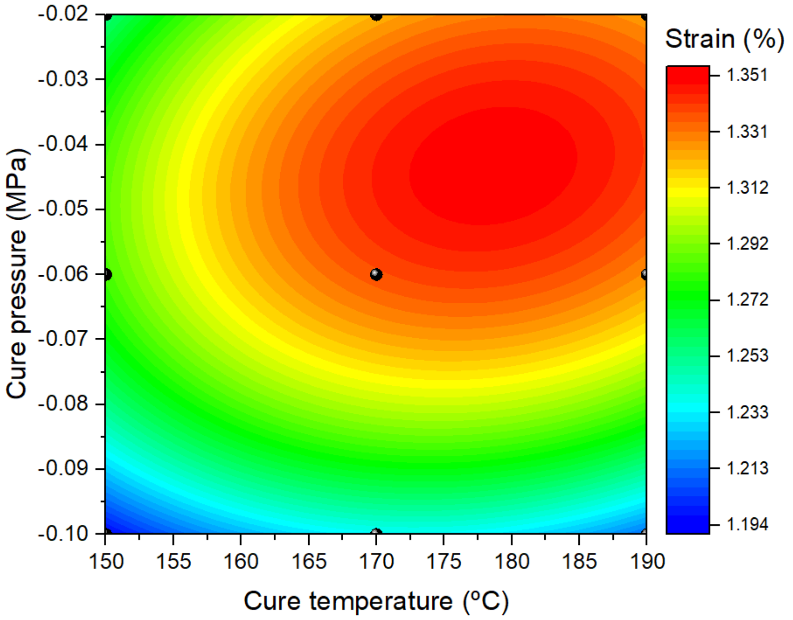


**Figure S34.** RSM of flexural strain as a function of cure pressure and temperature.

$$s = -1.4 + 0.03T - 6.1P - 7.9 \times 10^{-5}T^2 - 36.8P^2 + 0.02PT \quad (S30)$$

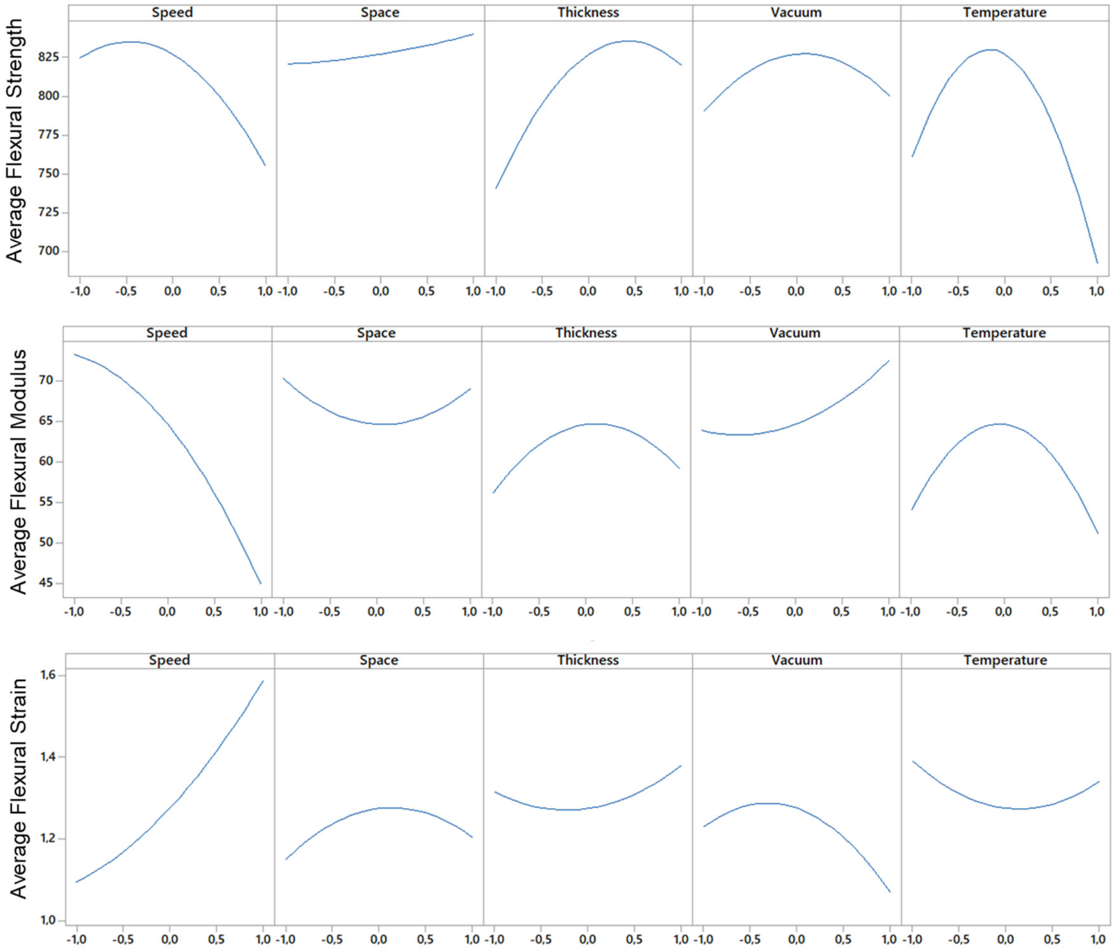


**Figure S35.** Relation of optimized parameters levels for flexural strength, modulus and strain.

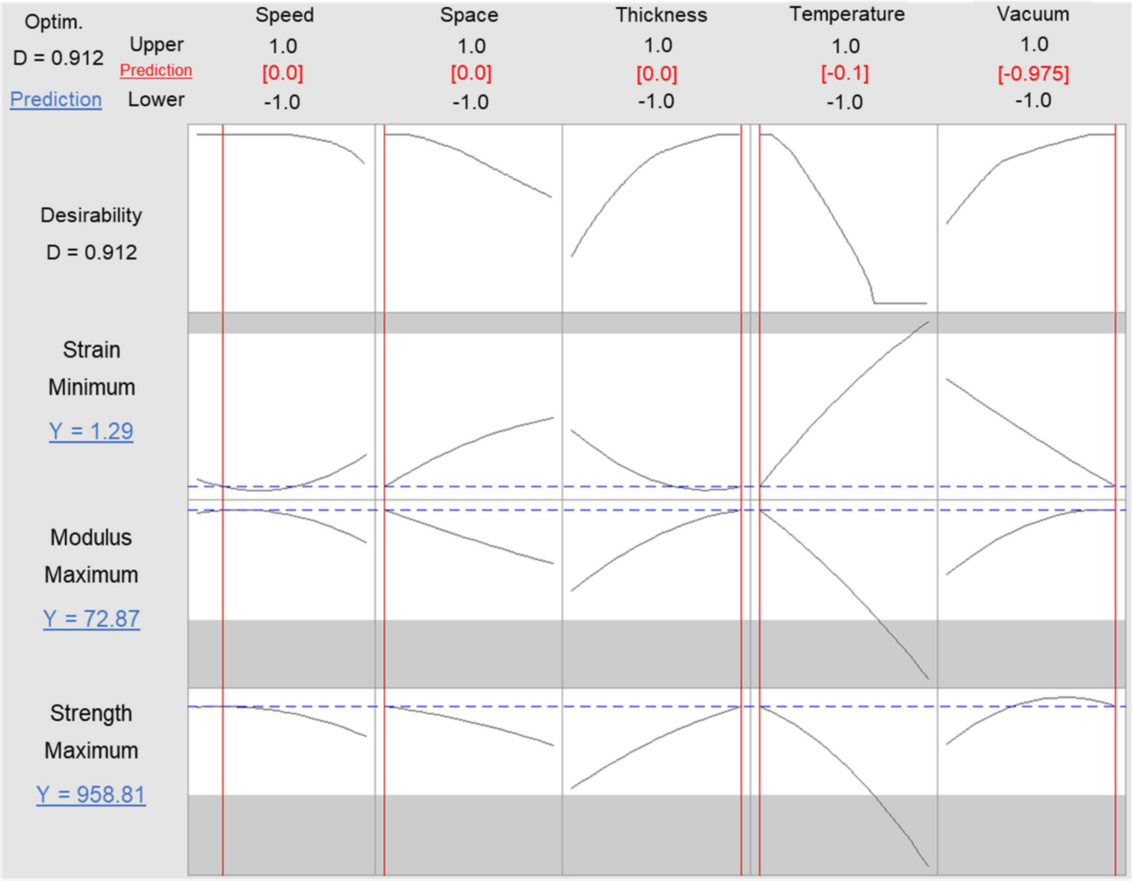


**Figure S36.** RSM Predicted mechanical properties based on processing parameters optimization (desirability diagrams).

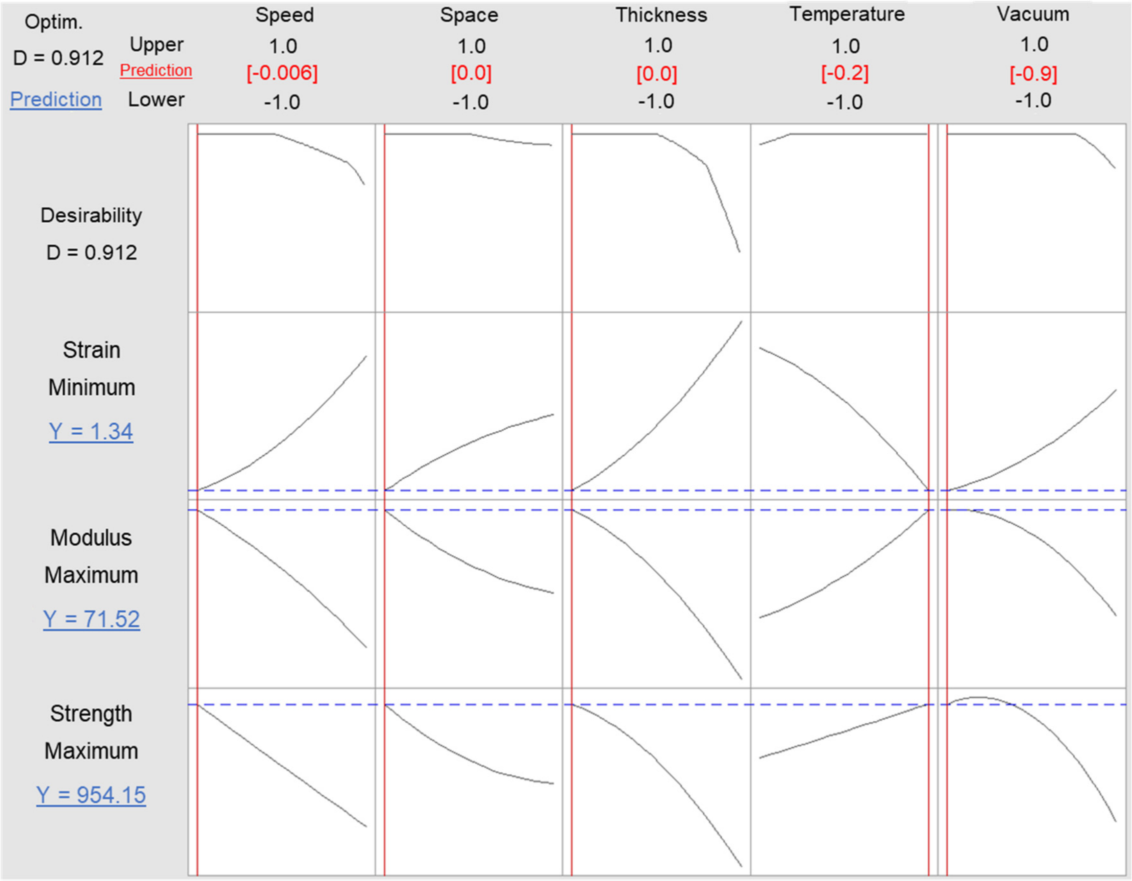


**Figure S37.** ANN Predicted mechanical properties based on processing parameters optimization (desirability diagrams).
